# Concepts of patients with alopecia areata about their disease

**DOI:** 10.1186/1471-5945-5-1

**Published:** 2005-01-12

**Authors:** Alireza Firooz, Mehdi Rashighi Firoozabadi, Behnaz Ghazisaidi, Yahya Dowlati

**Affiliations:** 1 Center for Research and Training in Skin Diseases and Leprosy, Tehran University of Medical Sciences, Tehran, Iran

## Abstract

**Background:**

Alopecia areata (AA) is a common and chronic skin disease with an unknown etiology. It may significantly affect the patient quality of life. This study was designed to evaluate the illness perception in patients with AA.

**Methods:**

A questionnaire consisting of 25 questions about causes, timeline, consequences and control of disease were given to 80 patients with AA attending a skin clinic in Tehran, Iran. The impact of age, gender, duration of disease, education, extent of disease and family history of AA were also assessed.

**Results:**

Eighty patients (38 male and 42 female) with a mean age of 27.5 years (SD = 9.3) and disease duration of 7.8 years (SD = 7.7) completed the questionnaire. 76.9% of the patients believed that the role of stress was the cause of disease. 17.1 % believed genetic background to be the main cause, this found to be more frequent in patients with positive family history of AA. More than half of patients believed that their illness had major consequences on their lives and 40% of patients believed that their illness would be likely to be permanent rather than temporary, more in patients with longer duration of disease. Only 57.5% of patients considered their treatments to be effective.

**Conclusion:**

AA may considerably affect various aspects of patients' lives. The patient knowledge about the causes and course of this disease is limited.

## Background

Alopecia areata (AA) is manifested as a sudden loss of hairs without any inflammation or scarring. The hair loss might be seen in a circumscribed area or the whole scalp (alopecia totalis or AT) or whole body (alopecia universalis or AU) [1]. It is a common disease and at any given time, 0.2% of the population has AA and 1.7% of the population will experience an episode of AA during their lifetime [2,3]. The etiology of AA is not known exactly. However factors such as genetic predisposition, autoimmunity, and stress have been suggested [4,5]. The course of disease is not predictable and it is often associated with periods of hair loss and regrowth. The clinical severity of a patient's AA may not be a good indicator of subsequent downturn in quality of life or psychological well-being.

The onset of a chronic condition brings with it a range of difficulties that may show considerable variation in their nature and severity as perceived by the patient. In order to make sense of and respond to the difficulties that chronic illness may present, patients construct their own common-sense cognitive model of their condition. Such models are based upon information received from a range of sources including their physician, family, friends, and existing social and cultural notions about health and illness. The resulting system of beliefs can of course be flawed or inaccurate; however there is evidence that it is those beliefs that drive attempts to cope with a condition and issues of compliance with treatment. Patient-held beliefs have important implications for the clinical management of their disease. Studies on patients with psoriasis and acne vulgaris have shown that knowledge of patients about their condition, the course of their disease and current treatments is not appropriate [6,7].

In this study, we examined the system of beliefs held by AA patients and the factors that might influence such beliefs.

## Methods

The Illness Perception Questionnaire (IPQ) [8] with a few modifications was given to 80 patients with AA older than 12 years, attending a private skin clinic in Tehran, Iran in 1999. The study was approved by Institutional Review Board of the Center for Research and Training in Skin Diseases and Leprosy. The IPQ was created to provide a theoretically derived measurement instrument suitable for use with any patient population. It has been used in patients with cardiac disease [9], chronic fatigue syndrome [10], diabetes, chronic pain, rheumatoid arthritis [8], and psoriasis [6]. As AA is an asymptomatic disease, we did not use the subscale of "symptoms" in our study. Thus the questionnaire that we used consisted of four subscales:

**Cause **subscale (10 items) measures personal ideas about the cause of AA.

**Time line **(3 items) deals with perceptions about how long the disease will last.

**Consequences **(6 items) are concerned with expected effects and outcomes of the illness.

**Cure/Control **(6 items) details beliefs about recovery from or control of the condition.

There were four possible answers for each item in the IPQ to be chosen by patients: I strongly agree, I agree, I do not know, I disagree.

Furthermore, some demographic information such as age, sex, family history of AA, and duration and extent of disease (alopecia areata or AT/AU), and level of education were obtained from the patients to evaluate their influence on patients' beliefs.

Statistical analysis was conducted by means of SPSS statistical software, version 11.0. Because the data were not normally distributed, nonparametric statistics were used. Correlations were processed by Spearman's rank correlation, and differences between means were computed by means of the Mann-Whitney U test. For simplicity of analysis and increasing the power of study, the answers of "I strongly agree" and "I agree" were grouped together and compared with the answers of "I don't know" and "I disagree" which were grouped together as "I do not agree". A p value of less than 0.05 was considered as significant.

## Results

A total of 80 patients with AA (38 male, 42 female), with a mean age of 27.5 years (SD 9.3, ranged from 13 to 56 years) were recruited to the study. The mean duration of illness was 7.8 years (SD 7.7, ranged from 1 month to 30 years). In 75% of patients, AA was patchy and it was totalis or universalis in 25%. Fifteen percent of patients had a positive family history of AA in their first degree relatives. Physicians were the main source of patients' information about their disease in 66.2% of them.

### Beliefs about cause

Table. 1 shows the percentage of patients "agreeing" with each **cause **item. A total of 76.9% of patients believed that stress was a major factor in onset of their illness and older patients were more likely to believe in this (p < 0.05). Patients who had a belief that their disease was a result of genetic factor were more likely to have a family history of AA and longer duration of the disease (P < 0.05). Younger patients and those with extensive disease (AT/AU) believed that their illness was because of chance or fate (P < 0.05).

**Table 1:** Beliefs about causes of alopecia areata (n = 80)

**Causes**	**Agree**	**Factors influencing beliefs**
Stress	76.9%	Older patients (p = 0.012)
My state of mind	59.2%	None
My own behavior	47.3%	None
Other people	34.2%	None
Chance or fate	31.1%	Younger patients(p = 0.021), extensive disease (AT/AU)(p = 0.030)
Diet	25.7 %	None
Pollution	24.3%	None
Germ or virus	21.9%	None
Genetic	17.1%	Family history of AA(p = 0.006), longer duration(p = 0.017)
Poor medical care	11.8%	None

### Beliefs about consequences

Majority of the patients (58.2%) believed that their illness had a major consequence on their lives, 53.8% of patients also felt that AA had strongly affected their self-esteem, and 50.6% considered AA as a serious condition. These believes were stronger in younger patients, and in patients who had the disease for a long time (p < 0.05). Table 2 shows the percentage of patients "agreeing" with each **consequence **item.

**Table 2:** Beliefs about consequences of having alopecia areata (n = 80)

**Beliefs**	**Agree**	**Factors influencing beliefs**
My disease has had a major consequence on my life.	58.2%	Younger patients(p = 0.012), longer duration(p = 0.014)
My disease has strongly affected the way I see myself as a person.	53.8%	Younger age at onset(p = 0.003)
My disease has strongly affected the way others see me.	51.3%	Younger age at onset(p = 0.022), longer duration(p = 0.012)
My disease is a serious condition.	50.6%	Younger age at onset(p = 0.003), younger patients(p = 0.013)
My disease has become easier to live with.	50.6%	None
My disease has serious economic and financial consequences.	27.8%	Younger age at onset(p = 0.015), longer duration(p = 0.010)

### Beliefs about recurrence or chronicity

Half of the patients believed whether their disease cleared, it would always come back and forty percent of patients believed that their illness would be likely to be permanent rather than temporary. They were more likely to have a longer duration of disease (p < 0.05). The minority of patients (25.0%) believed that their illness would last a short time.

### Beliefs about cure and control

More than 60% of patients believed that their behavior could determine improvement or worsening of their illness (table 3). This belief was present in female patients more than male patients (p < 0.05). 30.4% of patients believed there was very little that could be done to improve their illness. They were more likely to have longer duration of disease (P < 0.05). Thirty-eight percent of the patients believed that recovery from disease is largely dependent on chance or fate. This belief was stronger in female patients, those with younger age at onset, and patients with extensive disease (p < 0.05).

**Table 3:** Beliefs about cure and control (n = 80)

**Beliefs**	**Agree**	**Factors influencing beliefs**
What I do can determine whether my disease gets better or worse.	63.3%	Female patients(p = 0.010)
My treatment will be effective in curing my disease.	57.5%	None
My disease will improve in time.	53.2%	Older patients(p = 0.006), older age at onset(p = 0.029)
There is a lot that I can do to control my disease.	52.5%	None
Recovery from my disease is largely dependent on chance or fate.	38.0%	Female patients(p = 0.014), younger age at onset(p = 0.036), extensive disease (AT/AU)(p = 0.021)
There is very little that can be done to improve my disease.	30.4%	Longer duration(p = 0.004)

## Discussion

Alopecia areata is a chronic disease which may influence individual or social aspects of patients' lives. The results of our study confirmed this fact as the majority of patients believed that their illness had strongly affected their lives. It also influenced their self-esteem. The results of studies in other chronic diseases with periods of remission and exacerbation have had different results in this respect. For example in a study on acne patients, the disease had affected patients' self-image in nearly all of them, but it had no impact on interpersonal relationships, work, or school activities in majority of patients [7]. On the other hand, in a study on patients with psoriasis using the IPQ questionnaire, 68% of patients who suffered from psoriasis, agreed that psoriasis had a major consequence on their lives, and 53.4% agreed that psoriasis had strongly affected the way they saw themselves as a person [6].

The present study also investigated cognitive appraisals held by patients about their illness and showed that such beliefs were not associated in any significant manner with the extent of their condition. Fortune et al also did not find an association between the clinical severity of psoriasis and beliefs held by patients about their condition [6]. Thus the assumption that the objective severity of a condition will be associated in a linear fashion with patient's subjective experience in terms of beliefs, coping, or distress is unlikely to be correct. On the other hand, young patients and those with longer duration of disease were more likely to be affected by their disease. This implies that the chronicity of the disease has more influence on patient's life than the extent of it.

The results of our study also showed that the beliefs about the consequences of having AA were not influenced by the gender of the patients. Thus men are as vulnerable as women in suffering from the consequences of AA.

Patients with AA, including 77% of patients in this study, often attribute the onset of their disease to a specific stressful life event. In a study on 178 patients, Van der Steen *et al. *showed that emotional stress is not an important factor in the initiation of AA [11]. Brajac *et al. *did not find a significant role of stress in the onset of AA but stressful life events had an important role in triggering of some episodes of disease [12]. On the other hand, Gupta *et al. *found that AA patients who were depressed, were more likely to mention stress as the cause of their disease [13].

In recent studies, psychologic and psychopathologic factors have been analyzed as modulators of neuroendocrinologic, vascular, and immunologic variables; this is far from the initial concept of stress being the causal agent in the illness. In fact, stress may cause its effect by making alterations in immune responses related to neuropeptides, such as the migration of the macrophages, vasodilator or vasoconstrictor responses, phagocytosis, lymphocytic cellular immunity, and expression of some factors of leukocytic adhesion to the microvascular endothelium [14]. In addition, the adaptation to the illness is regarded as an important factor with regard to prognosis. However the exact cause of AA is not known and such events are very common, making it difficult for the investigator to prove that they are in fact involved in causing or precipitating the disease. In this study, one-third of the patients believed in chance or fate as the cause of AA, and this belief was stronger in younger patients and those with extensive disease (AT/AU).

This study also showed that as AA lasts, the patients feel more hopeless about time line and treatment modalities of their disease. The majority of patients had no hope to get rid of their disease. Almost half of patients expected their disease to relapse after it disappeared. Such perpetual stresses are hardly endurable. The psychiatrists' intervention may alleviate patients' stress and improve their quality of life.

This study was performed in a private dermatology clinic. The possibility of socioeconomic homogeneity among recruited patients may be biasing the results. So it should be considered that these results may be different in patients with AA in different socioeconomic and cultural backgrounds.

## Conclusion

There is a need for accessible, accurate, community-based education on the natural history of AA, the effectiveness and expected duration of treatment. The inadequacy of information provided by current sources is evident in ongoing misconceptions on causality and the perceptions of respondents. Incorporating information on this disease may facilitate patient into therapeutic selection, enhance understanding of treatment options and improve patient compliance.

## Competing interests

The author(s) declare that they have no competing interests.

## Authors' contributions

AF participated in the design and conduct of the study and preparation of the manuscript.

MRF participated in the conduct of the study and statistical analysis.

BG participated in the conduct of the study and statistical analysis.

YD participated in the design of the study and preparation of the manuscript.

All authors read and approved the final manuscript.

## Pre-publication history

The pre-publication history for this paper can be accessed here:


